# Up-Regulating CYP3A4 Expression in C3A Cells by Transfection with a Novel Chimeric Regulator of hPXR-p53-AD

**DOI:** 10.1371/journal.pone.0095752

**Published:** 2014-05-01

**Authors:** Feng Chen, Xiao-Hui Rao, Jin-Lian Yang, Ming-Xing Pan, Yi Gao, Zhen-Lin Li, Yang Li, You-Fu Zhu, Yan Wang

**Affiliations:** 1 Institute of Regenerative Medicine, Southern Medical University Zhujiang Hospital, Guangzhou, China; 2 Department of Hepatobiliary Surgery, Huizhou Municipal Central Hospital, Huizhou, China; 3 Department of Hepatobiliary Surgery, Southern Medical University Zhujiang Hospital, Guangzhou, China; 4 Department of Histology and Embryology, Southern Medical University, Guangzhou, China; 5 Department of Infectious Diseases, Southern Medical University Nanfang Hospital, Guangzhou, China; Northeast Ohio Medical University, United States of America

## Abstract

Most hepatoma cell lines lack proper expression and induction of CYP3A4 enzyme, which limits their use for predicting drug metabolism and toxicity. Nuclear receptor pregnane X receptor (PXR) has been well recognized for its critical role in regulating expression of CYP3A4 gene. However, its physiological activity of binding to the particular site of promoter is significantly weakened in hepatic cell lines. To address this problem, we created “chimeric PXR” constructs by appending a strong activation domain (AD) from p53 subunit to either N- or C- termini of the human PXR (hPXR), that is, hPXR-p53 and p53-hPXR. C3A, a hepatoma cell line, was used as the cell model to test the regulation effect of chimeric hPXR over wild type (WT) hPXR on CYP3A4 expression at gene, protein, and metabolism levels, respectively. Compared with C3A cells transiently transfected with WT hPXR, the activity of CYP3A4.XREM.luc reporter gene in C3A cells transfected with hPXR-p53 or p53-hPXR increased 5- and 9-fold respectively, and the levels of CYP3A4 mRNA expression increased 3.5- and 2.6-fold, respectively. C3A cells stably transfected with hPXR-p53-AD exhibited an improved expression of CYP3A4 at both gene (2-fold) and protein (1.5-fold) levels compared to WT C3A cells. Testosterone, a CYP3A4-specific substrate, was used for detecting the metabolism activity of CYP3A4. No testosterone metabolite could be detected in microsomes from WT C3A cells and WT C3A cells-based array, while the formation of 6β-hydroxytestosterone metabolite in the transfected cells was 714 and 55 pmol/mg protein/min, respectively. In addition, all the above expression levels in the transfected cell models could be further induced with additional treatment of Rifampicin, a specific inducer for CYP3A4. In conclusion, our study established a proof-of-principle example that genetic modification with chimeric hPXR-p53-AD could improve CYP3A4 metabolism ability in hepatic cell line.

## Introduction

Cytochromes P450 (P450s or CYPs) are a heme-thiolate monooxygenases that play an important role in the metabolism of drugs. Human CYP3A family consists of the subtypes CYP3A4, CYP3A5, CYP3A7, and CYP3A43 [1]. These enzymes are ample in human liver, and CYP3A4 is the most important and abundant one [2]. CYP3A4 has a wide spectrum of metabolism substrates; its importance in drug metabolism is highlighted by the fact that it contributes to the metabolism of approximately 60% of marketed drugs [3]. Because of the great impact of CYP3A4 on efficacy and toxicity of new drugs, *in vitro* metabolic experiments with primary hepatocyte or hepatoma cell lines are used to assess and predict xenobiotic metabolism or toxicity at an early stage of drug development.

In cell models for drug testing, primary human hepatocytes remain the standard method, even though they have well-known limitations including poor availability, batch-to-batch variability, non-proliferation in culture and severe phenotypic function drop-off, such as the rapid loss of CYPs activity, whatever systems or conditions are taken for *in vitro* culture [4]–[6]. As a practical alternative, hepatoma cell lines are used with evident advantages with respect to their availability and relatively stable phenotype between appropriate generations; however, they express CYP enzymes at much lower levels compared to their primary counterpart [7]. Different strategies to up-regulate expression level of drug-metabolizing enzymes have been used with aim to generate primary hepatocyte-mimicing systems. For instance, hepatoma cells were treated with CYP-inducing chemicals such as vitamin D or dexamethasone [8], or stably transfected with liver-specific transcription factors such as CCAAT/enhancer-binding protein α (C/EBPα) or with individual CYP constructs [9]–[11]. However, the improved expression level of CYP genes initiated by these strategies only begins to approach that of primary hepatocytes, which are themselves significantly lower than fresh tissue [12].

Pregnane X receptor (PXR) regulates the expression of many genes involved in xenobiotic metabolism [13]–[15]. Its target genes include CYP3A4, CYP2B6, CYP2C subfamily, several conjugation enzymes and drug transporters as well [16], [17]. Therefore, cell lines were treated with PXR agonists or transfected with PXR expression vector to increase expression of several CYP mRNAs [18]. The potential advantage here is that several PXR-target genes may be up-regulated at the same time only by introducing the sole PXR construct. However, the consequence of trans-activation of PXR has often been moderate in various reporter gene assays [19] and the up-regulation of endogenous CYP3A4 or CYP2B6 mRNAs has been quite modest [18]. These findings indicated the limitation of transcriptionally regulating CYP genes by introducing a native PXR into hepatoma cell lines.

Inspired by the function-modular structure of PXR [20], some studies tried to append PXR molecule with an extra heterogeneous strong AD, with expectation to enhance the trans-activation mediated by PXR. For example, transgenic mice were generated carrying fusion of the hPXR cDNA with the AD from the herpes simplex viral protein 16 (VP16-AD) [14], which had been used to form an ecdysone-inducible regulator for gene therapy and cell biological studies [21]. Due to the constitutive activity of VP16-AD fusion partner, these transgenic mice showed up-regulation of PXR-target hepatic genes without any exposure to PXR ligand. Similarly, the AD of p65 subunit of nuclear factor κB (p65-AD) has been applied to form a nuclear receptor-based regulator for *in vitro* cell model for drug and xenobiotic metabolism [22]. Based on these previous reports, we proposed that trans-activation effect of hPXR may be enhanced by fusion with a heterologous AD, and subsequently, the ability of these “chimeric hPXR” to activate CYP3A4 gene in hepatoma cells would be higher as compared to native hPXR; thus, chimeric hPXRs might be effective in producing hepatoma cells with an increased expression of CYP3A4.

In our study, C3A cell line, a clonal derivative of hepatoblastoma-based HepG2, was employed as cell model due to its relatively higher-level of remnant hepatic phenotype and popularity in drug testing [23]. Chimeric hPXRs were designed by fusing hPXR with p53-AD either at its N- or C-terminus. They were then characterized by measuring reporter gene activity and their effects on CYP3A4 transcript levels in C3A cells. Finally, a cell line stably expressing hPXR-p53-AD was selected by measuring the expression of CYP3A4 mRNA and analyzed for its CYP3A4 expression at protein levels, and drug metabolism activity.

## Materials and Methods

### Chemicals

Rifampicin (RIF) and dimethylsulfoxide (DMSO) were purchased from MP Biomedicals (Solon, OH, USA). Testosterone was from Sinopharm Chemical Reagent Co., Ltd (Shanghai, China). 6β-hydroxytestosterone was from Cayman Chemical (Ann Arbor, MI, USA).

### Construction of Wild-type and Chimeric hPXR Expression Vectors

**Figure 1:**
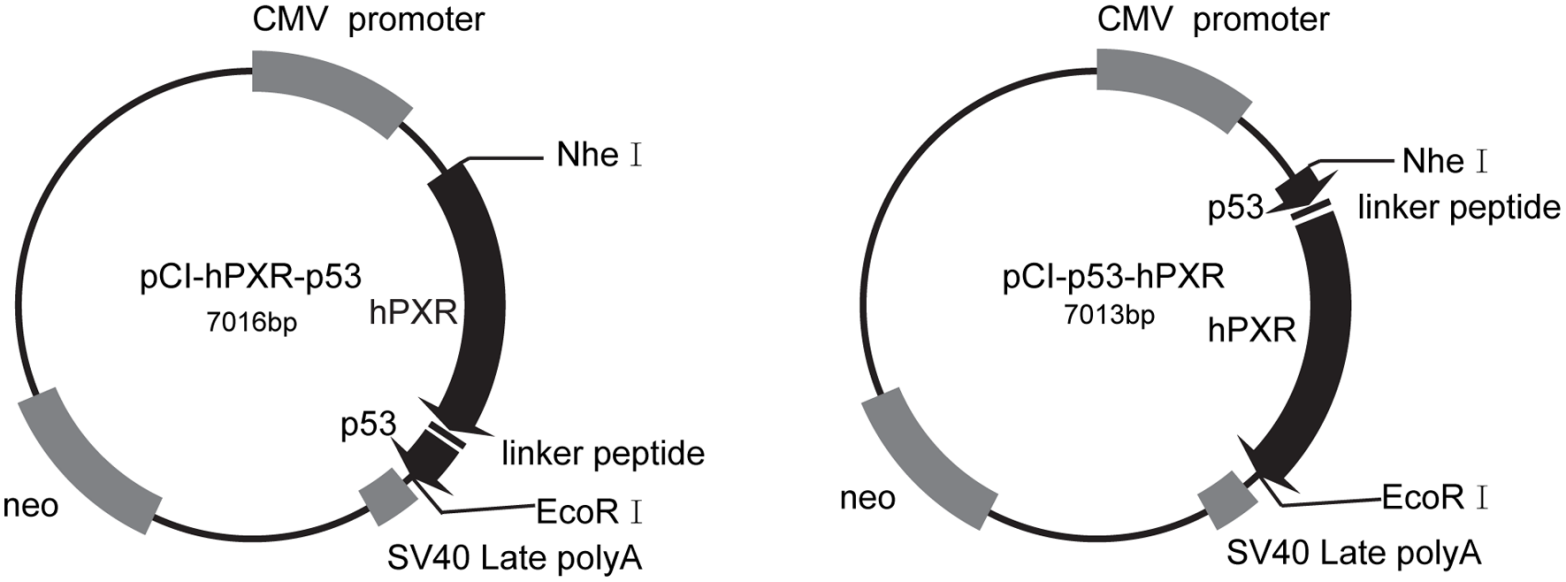
Schematic illustration of chimeric hPXR constructs designed in the present study. CMV promoter = cytomegalovirus immediate-early enhancer/promoter region; SV40 late polyA = simian virus 40 late polyadenylation signal.

The full-length of WT hPXR (residues 1–434) expression vector has been described in previous reports [24], [25]. **Fig. 1** showed the schematic structure of chimeric hPXR constructs that were cloned into a CMV-driven pCI-neo expression vector (Promega, Madison, WI, USA). The region containing a nuclear localization signal and an AD fragment [26] of human p53 was amplified from a human liver (HL) tissue-derived cDNA pool using a mixture of dNTP, pfx DNA polymerases and specific primers (**Table 1**). To construct N-terminal chimeric plasmid pCI-p53-hPXR, the p53 fragment was fused in-frame with sequences encoding a linker peptide GSTRYQA and the full-length hPXR, respectively. To construct C-terminal chimeric plasmid pCI-hPXR-p53, the full-length hPXR cDNA was fused in-frame (with deletion of the hPXR stop codon) with sequences encoding a linker peptide GSTRYQAR, the p53 fragment and a new stop codon, respectively. A detailed description of the cloning procedure is available upon request.

**Table 1:** Primers’ sequence used for qPCR.

Gene	Sequence
18S rRNA	F: CCTGGATACCGCAGCTAGGA
	R: GCGGCGCAATACGAATGCCCC
CYP3A4	F: AAAGTCGCCTCGAAGATACA
	R: GAGAACACTGCTCGTGGTT
p53 of hPXR-p53	F: GGTCGGATCCACACGATACCAAGCTCGCGAGGAGCCGCAGTCAGATCC
	R: GGTGGAATTCTTACACGGGGGGAGCAGCC
p53 of p53-hPXR	F: GGTCGCTAGCCACCATGGAGGAGCCGCAGTCAGATC
	R: GGTGGGATCCCACGGGGGGAGCAGCCTC

Sequence direction: from 5′ to 3′. hPXR = human pregnane X receptor; F = forward primer; R = reverse primer.

### Construction of Reporter Genes

Human CYP3A4.XREM.luc reporter containing a proximal 362 bp of CYP3A4 gene promoter and a distal enhancer was constructed following the method described previously [9]. The resulting construct containing two fragments of CYP3A4 5′- flanking region (−7836 to −7208 and −362 to +53) was linked to luciferase reporter sequence of pGL3-basic vector and hereafter designated CYP3A4.XREM.luc. Correct insert orientation was confirmed by DNA sequence analysis. pRL-TK vector was purchased from Promega (Madison, WI, USA).

### Cell Culture and Transfection

C3A (ATCC CRL-10741) and HEK293T (ATCC CRL-11268) cells were both respectively grown on 25 cm^2^ flasks at 37°C in 5% CO_2_ and passaged once a week. Standard growth medium used was Dulbecco’s Modified Eagle medium (HyClone, Logan, UT, USA), supplemented with 10% fetal bovine serum (Hyclone, Logan, UT, USA), 4 mM L-glutamine and 100 U/ml penicillin–100 mg/ml streptomycin. Culture medium was refreshed every 2 to 3 days. Passages between 3 and 30 were used for transfections.

Cells were seeded on 6-well plates (600,000 cells/well) one day before the transfection. Cells were transfected (4 µg DNA per well) with WT or chimeric hPXR expression vectors using lipofectamine 2000 (Invitrogen, Carlsbad, CA, USA). Standard growth medium in the absence or presence of RIF was added to the cells transfected with WT or chimeric hPXR expression vectors and the cells were incubated for 48 h before measurement of CYP3A4 mRNA.

### Reporter Gene Assays

Reporter gene assays were performed with a dual luciferase reporter assay kit (Promega, Madison, WI, USA). Cells were seeded on 96-well plates at approximately 90% confluency and co-transfected as described before [25] with various hPXR constructs (46 ng), CYP3A4.XREM.luc reporter (140 ng) using the Lipofectamine 2000 (Invitrogen, Carlsbad, CA, USA). All experiments for luciferase assay were performed by co-transfection of pRL-TK (Promega, Madison, WI, USA) renilla luciferase (14 ng) as an internal control for transfection efficiency. After 4–6 hr of transfection, culture medium was changed. At 24 hr after transfection, cells were harvested and luciferase activities were measured by a Modulus Single Tube Multimode Reader (Turner Biosystems, Sunnyvale, CA, USA). Each experiment was performed in triplicate, and the data was represented as mean±S.D. of three independent experiments after normalization to Renilla activity.

### Development of Stably Transfected Cell Lines

WT C3A cells were transfected with chimeric hPXR expression vectors using Lipofectamine 2000 (Invitrogen, Carlsbad, CA, USA), and growth medium supplemented with 0.6 to 0.8 mg/ml G418 (Calbiochem, San Diego, CA, USA) was used for selection as described before [27]. Proliferating G418-resistant colonies were picked and further cultured on 6-well plates in the presence of 0.4 mg/ml G418. The best colony was selected out with reference firstly to the results of qPCR for CYP3A4 mRNA and subsequently to CYP3A4 protein expression level, as depicted in qPCR and Western blot analysis sections, respectively.

### Quantitative RT-PCR Luminometer Luminometer

Cells were seeded on 6-well plates until confluent. A 1-ml aliquot of TRIzol reagent (Molecular Research Center, Inc., Cincinnati, OH, USA) was added to each well, and total RNA was extracted in accordance with the manufacturer’s instructions. The content of total RNA was determined at 260 nm, and its purity was estimated using a ratio of 260/280 nm. One microgram of total RNA was reverse-transcribed by M-MLV enzyme (Promega, Madison, WI, USA) to cDNA according to the manufacturer’s protocol.

For normalization of the results, 18S rRNA was used as the internal control. cDNA was amplified for 40 cycles with an ABI Prism 7500 Sequence Detection System (Applied Biosystems, UK). The primer sequences used are listed in **Table 1**. Quantification was performed using the 2^−ΔΔCT^ method. The comparative threshold cycle (C_T_) method was used to calculate the relative expression. For quantification of gene expression, the target gene values were normalized to the expression of endogenous reference (18S rRNA). The amount of target relative to a calibrator (normal pool expression) is given by 2^−ΔΔCT^ [ΔC_T_ = C_T_ (target gene) - C_T_ (18S rRNA); ΔΔC_T_ = ΔC_T_ for any sample - ΔC_T_ for the calibrator].

### Western Blot Analysis

Cells were grown on 100 mm plates until confluent, and then washed and lysed in lysis buffer supplemented with protease inhibitors. HL tissue, as a positive control, was isolated from resected normal human liver tissue. Protein concentrations of the lysates were measured using the BCA assay kit (Pierce, Rockford, IL, USA). The lysates were subjected to SDS-polyacrylamide gel electrophoresis using a 10% gel, then transferred to a PVDF membrane (Millipore, Bedford, MA, USA). After blocking in 5% (w/v) fat-free milk powder, the membranes were incubated with rabbit anti-human CYP3A4 polyclonal antibody (AB1254; 1 : 2000; Millipore, Temecula, CA, USA), or rabbit anti-β-actin antibody (E12-051-1; 1 : 3000; EnoGene, New York, NY, USA), followed by goat anti-rabbit conjugated horseradish peroxidase secondary antibody (bs-0295G-HRP; 1 : 5000; Bioss, Beijing, China). The proteins were visualized with an enhanced chemiluminescence detection system (Pierce, Rockford, IL, USA). The relative amounts of CYP3A4 protein were estimated from densitometric analysis of the blot after scanning (Adobe Photoshop 3.0; Adobe System, Mountain View, CA, USA). β-actin was used as a loading control.

### Determination of CYP3A4 Enzyme Activity

6β-Hydroxylation of testosterone was used as a marker of functional CYP3A4 expression as described previously [28]. Microsomes were prepared as described [29], [30] from cells in absence or presence of RIF. In brief, cells were harvested after washing twice with ice-cold phosphate-buffered saline. They were pelleted by centrifugation at 1,000 rpm for 5 min, and then homogenized with a glass homogenizer in 0.25 M sucrose, 1 mM EDTA, 1 mM DTT, 50 mM potassium phosphate buffer (pH = 7.4) and 1 mM PMSF on ice. The homogenate was first centrifuged at 10,000×g for 15 min at 4°C. The resulting supernatant was centrifuged at 100,000×g for 1 hr, and the obtained microsomal pellet was resuspended in 50 mM potassium phosphate buffer, pH 7.4. Protein concentration was determined by the Bradford assay with bovine serum albumin as the standard. The reaction mixture (total volume, 0.5 ml) contained 100 µg of microsomes, 200 µM testosterone, 50 mM potassium phosphate buffer, pH 7.4, and 30 mM magnesium chloride. After 5 min pre-incubation, the reaction was initiated by the addition of 5 mM NADPH, and after 20 min incubation at 37°C, the reaction was stopped with 2.5 ml of ethyl acetate. The content of testosterone metabolite 6β-hydroxytestosterone was analyzed using high performance liquid chromatography (HPLC) as depicted in HPLC analysis section.

Meanwhile, the cell-based assay was used to test CYP3A4 catalytic activity. Cells were treated with CYP3A4-specific substrate testosterone. One milliliter of incubation medium (without phenol red) containing 200 µM testosterone was added concomitantly to each well and incubated for 3 hr at 37°C. The medium was collected and stored at −20°C until analysis. Cells were lysed with 1% Triton X-100 in phosphate-buffered saline, and protein concentration in each well was determined using the BCA assay kit (Pierce, Rockford, IL, USA). For induction studies, 1 ml of induction medium containing 20 µM RIF was added to the cells and replaced with fresh induction medium after 24 hr. After another 24 hr, 1 ml of medium containing 200 µM testosterone was added as described above. The content of testosterone metabolite 6β-hydroxytestosterone was analyzed using HPLC as described in HPLC analysis section.

### HPLC Analysis

Each sample was centrifuged for 10 min at 13,000× g and 20 µl of the supernatant containing 96 µM 4-androsterone-3,17-dione (as an internal standard) was analysed using an Agilent 1100 HPLC system equipped with an auto-sampler. The samples were separated on a reversed-phase C_18_ column (5 µm, 250 mm×4.6 mm; Elite, Dalian, China) with a binary gradient of mobile phase A and B (55% methanol/45% water, v/v). The mobile phase flow rate was 1 ml/min, and the column was maintained at 38°C. Column effluent was then monitored by ultraviolet absorbance at 254 nm. The column was re-equilibrated for 2 min before injecting the next sample. Finally, the peak areas of internal standard and metabolite were extracted from the chromatograms and were used to quantify the 6β-hydroxytestosterone.

### Statistical Analysis

GraphPad Prism 5.0 was used for the drawing and statistical analysis. Data were analyzed using one-way analysis of variance (Dunnett’s multiple comparison test) or two-way analysis of variance (Bonferroni post-tests), and *p* value<0.05 was considered to be significant. Data are expressed as mean±S.D. of three independent repeats.

## Results

### Construction of Chimeric hPXR Expression Vectors

To validate if the chimeric hPXR expression vectors were constructed as designed, we detected nucleotide sequence of the chimeric hPXRs. After digestion by restriction endonucleases *Nhe I* and *BamH 1*, the pCI-hPXR-p53 construct would be splitted into three fragments, and their sizes were 1.3 kb, 2.5 kb and 3.2 kb, respectively. After digestion by restriction endonucleases *Nhe I* and *EcoR I*, the pCI-p53-hPXR construct would be divided into two fragments (1.6 kb and 5.4 kb). The digested constructs were subjected to agarose gel electrophoresis, and the constructs conformed to the above demands were then checked by DNA sequencing (for more details, see figure S1). DNA sequencing results showed that the chimeric hPXRs were successfully constructed (for more details, see figure S2-S3).

### Chimeric hPXR Constructs Increased the Activity of CYP3A4.XREM.luc Reporter Gene

**Figure 2:**
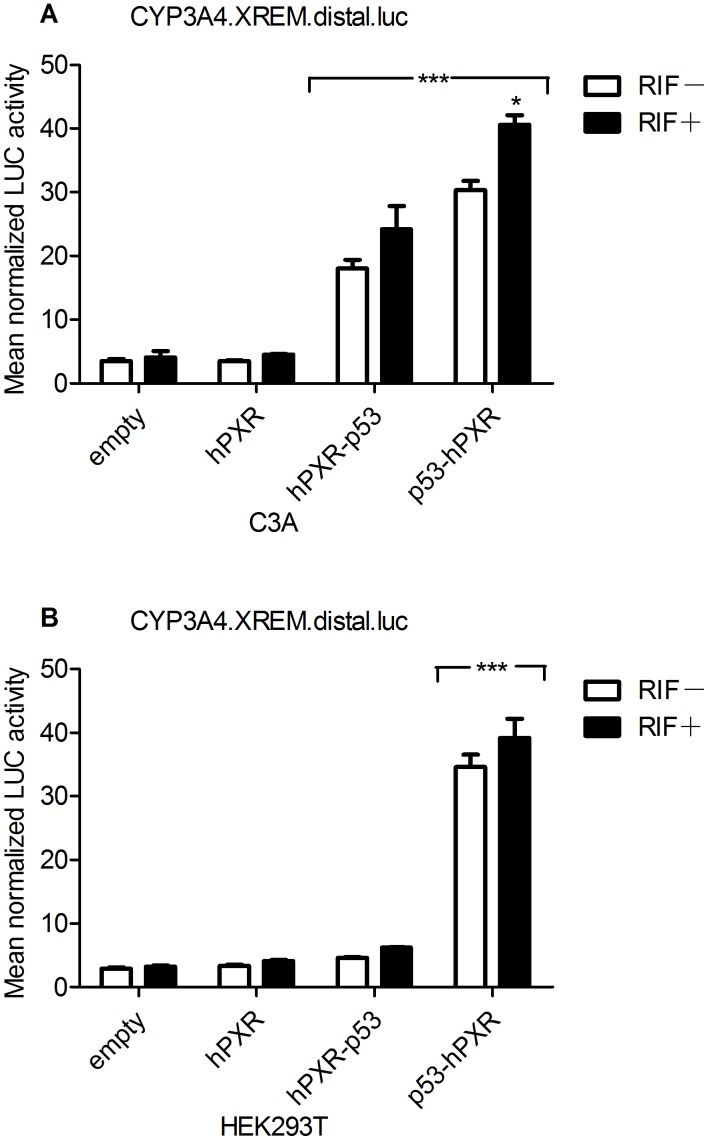
Activation of CYP3A4.XREM.luc luciferase reporter by various hPXR constructs in WT C3A and HEK293T cells. The empty vector contained the pCI-neo plasmid instead of hPXR construct. The ligand (black columns) was 20 µM RIF while vehicle (white columns) was 0.02% (v/v) DMSO. The ratio of reporter luciferase activity to control renilla luciferase activity is indicated. Results are expressed as the mean±S.D. of normalized luciferase activity of three independent measurements. Statistical significance p<0.05: *, RIF-treated vs. respective DMSO control; ***, chimeric hPXR vs. WT hPXR vector.

To study if chimeric hPXRs can activate the promoter of CYP3A4 gene as its WT counterpart does, we investigated the effect of different hPXR constructs on CYP3A4.XREM.luc reporter gene in C3A cells (**Fig. 2A**). C3A cells were transiently co-transfected with chimeric hPXR constructs and CYP3A4.XREM.luc reporter gene, in comparison with WT hPXR construct; and then we tested their response to CYP3A4 inducer, RIF. In absence of RIF, CYP3A4.XREM.luc was strongly activated by chimeric hPXRs, with the highest increase being close to 9 folds over the WT hPXR. Upon the addition of RIF, the reporter activity was clearly increased further.

To further verify the activation specificity, we employed HEK293T, a non-hepatic cell line, to observe the effect of chimeric hPXRs transfection in the same way as we did with C3A. It was found that WT hPXR did not appreciably affect the basal CYP3A4.XREM.luc reporter activity in absence of RIF. In contrast, chimeric hPXRs with N- or C- terminal p53-AD displayed a strong constitutive activation of the CYP3A4 reporter (about 10 or 1.4 folds, respectively) (**Fig. 2B**).

The results collectively indicated that chimeric hPXRs in our study performed a stronger activation of CYP3A4 promoter than WT hPXR did, in a specific and physiology-like manner.

### Transient Transfection of Chimeric hPXR Constructs Significantly Improved CYP3A4 mRNA Expression

**Figure 3:**
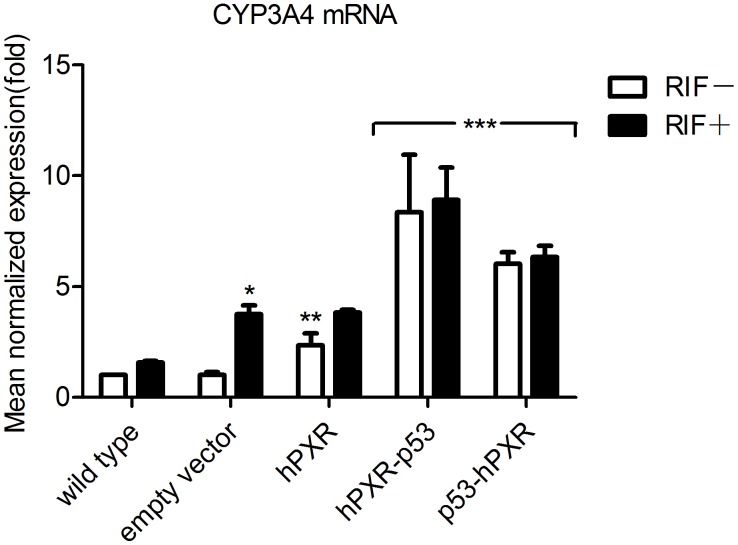
CYP3A4 mRNA expression in C3A cells transiently transfected with different hPXR constructs. The relative mRNA levels compared with mRNA levels in the WT C3A without the addition of RIF control cells (relative expression value set to 1) were defined by the 2^−ΔΔCT^ method. Results are expressed as mean±S.D., n = 3. Statistical significance p<0.05: *, RIF-treated vs. respective DMSO control; **, WT hPXR vs. empty vector; ***, chimeric hPXR vs. WT hPXR vector.

To further investigate the characteristics of chimeric hPXRs, C3A cells were transiently transfected with WT or chimeric hPXR expression vectors to study their effects on expression of CYP3A4 mRNA (**Fig. 3**). Cells transfected with WT hPXR enhanced CYP3A4 mRNA expression 2.3-fold as compared to non-transfected cells. As expected, subsequent RIF treatment elevated the mRNA levels further by about 60%. These results indicate that over-expression of the full-length hPXR can, to some extent, activate the genomic copy of CYP3A4. As shown in **Fig. 3**, chimeric hPXRs can efficiently up-regulate expression of CYP3A4 mRNA from 6- to 8- fold when compared to the WT C3A. These results were superior to that of transfection of native hPXR construct and subsequent treatment with RIF, and proved that chimeric hPXRs could effectively reactivate CYP3A4 gene expression in C3A cells.

### C3A Stably Transfected with Chimeric hPXR-P53-AD Construct has Physiologically Improved CYP3A4 Expression at Gene, Protein and Activity Levels

**Figure 4:**
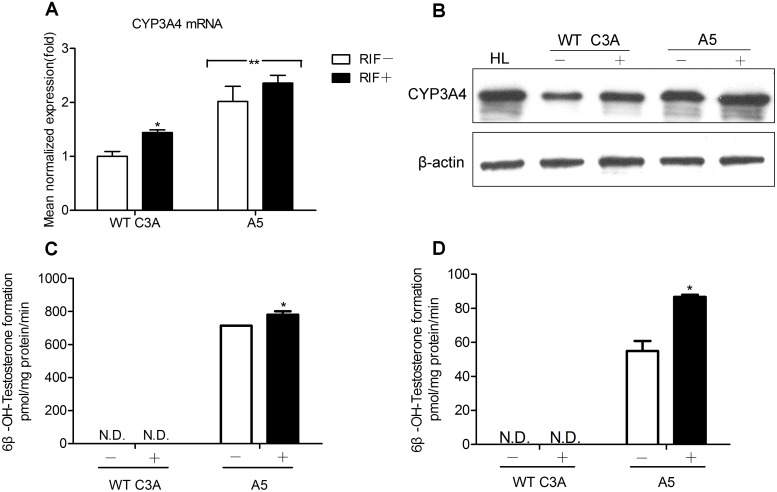
C3A stably transfected with chimeric hPXR-p53-AD construct (A5) has improved CYP3A4 expression at gene, protein and activity levels. (A) CYP3A4 mRNA expression in WT C3A and A5. The relative mRNA levels compared with mRNA levels in the WT C3A without the addition of RIF control cells (relative expression value set to 1) were defined by the 2^−ΔΔCT^ method. (B) The expression of CYP3A4 protein increased in A5 cells compared to WT C3A cells. Total proteins were isolated from WT C3A and A5 cells and analyzed by Western blotting. β-actin was used as a gel loading control. (C) CYP3A4 catalytic activity was determined by monitoring the 6β-hydroxylation of CYP3A4-dependent testosterone in microsomes isolated from A5 or WT C3A cells, as measured by HPLC. (D) CYP3A4 catalytic activity was determined by monitoring the formation of 6β-hydroxytestosterone in the medium of WT C3A or A5 cells, as measured by HPLC. For induction studies, 20 µM RIF was added 48 h before measurement. Data are expressed as mean±S.D. of three independent experiments. Statistical significance p<0.05: *, RIF-treated vs. respective DMSO control; **, A5 vs. WT C3A. N.D., not detected.

C3A cells were then transfected with chimeric hPXRs, and the stable transformants were selected with G418. Proliferating colonies were expanded and had their PXR mRNA expression detected to demonstrate the successful over-expression of chimeric hPXR constructs (for more details, see figure S4). Then they were screened for functional expression of chimeric hPXRs by CYP3A4 mRNA level analysis in comparison with WT C3A cells (**Fig. 4A**). Variation between colonies was significant, ranging from values below C3A cells to 2-fold higher expression (A5). This indicates that random integration of transgene and other endogenous factors have intensive influence on the expression performance of CYP3A4 gene. Finally, the colony A5 was selected for further analysis because of its elevated expression of CYP3A4 mRNA (>2-fold).

Increased CYP3A4 mRNA expression in A5 was corroborated by Western blot analysis, where a clearly increase (1.5-fold) in CYP3A4 protein was observed compared with WT C3A. Only low levels of CYP3A4 protein could be detected in control cells, while the protein levels of A5 cells were relatively comparable with those observed in HL. As a gel loading control, the protein levels of internal protein β-actin were used and shown at the bottom of the **Fig. 4B**. It is notable that colony A5 exhibited highly elevated expression of CYP3A4 protein.

We further determined the CYP3A4 metabolism activity of A5 cells. First, we detected the formation of 6β-hydroxytestosterone, the metabolite of CYP3A4-specific substrate testosterone, using microsomal fractions isolated from A5 and WT C3A cells. Although the concentration of 6β-hydroxytestosterone was beyond the lowest limit of detection in microsomes from WT C3A cells, there was a dramatic increase in the formation of 6β-hydroxytestosterone (714 pmol/mg protein/min) in the microsomes from A5 cells (**Fig. 4C**), confirming the previous results that these cells have functional CYP3A4 expression. Then, using a cell-based assay for the same substrate drug, we found no testosterone metabolite could be detected in WT C3A cells; but a significant increase of metabolite 6β-hydroxytestosterone formation (55 pmol/mg protein/min) was observed in A5 cells (**Fig. 4D**). Moreover, the CYP3A4 activity was significantly induced when the cells were treated with RIF.

## Discussion

CYP3A4, mainly regulated by PXR, is the most important drug-metabolizing enzyme, but its expression level in hepatic cell lines is considerably low, mainly because PXR’s physiological activity of binding to the particular site of CYP3A4 promoter is significantly weakened in such cells. In this study, we designed chimeric hPXR constructs to improve the trans-activation potency of PXR. Within the hepatic C3A cell-transfection model, the chimeric hPXRs performed a stronger activation of reporter gene compared to the native hPXR, and increased the expression of CYP3A4 in the cells.

As we know, the positionally conserved hydrophobic residues shared with VP16 and other transactivators are essential for transactivation. Like VP16 and other transactivators, the N- terminal AD of tumor suppressor protein p53, a potent transcriptional activator, possesses several conserved hydrophobic residues, and being similar in size, net negative charge, and transactivating potency to the well-defined AD of VP16 [31]. Thus, p53-AD was designed and applied in our study over VP16-AD due to the reported toxicity of the latter in some cell lines [27]. To our knowledge, p53-AD has not been reported before to be used in a chimeric regulatory molecule to enhance its DNA-binding and activation. Due to various activities of p53 in multiple signal pathways underlying cell behaviors, we preliminarily observed the expression levels of some involving genes that might be affected. The results showed that introduction of p53-AD had no effect on the expression levels of these genes such as GADD45A and BAX (for more details, see figure S5). In addition, we also found that transfection of chimeric hPXRs increased expression level of CYP2B6 mRNA in C3A cells (for more details, see figure S6), which corresponded to the knowledge that PXR controls the transcription of a batch of downstream genes other than CYP3A4 [32].

PXR is inherently expressed in hepatic cells such as C3A, but not in HEK293T cells. Therefore, in the reporter gene assays, HEK293T cells were employed in comparison with C3A to verify that different responses of the reporter gene were specially due to the effect of transfected vectors but not the inherent relevant regulators. In **Fig. 2B**, when inducing with RIF, the activity of reporter gene was not apparently increased in HEK293T cells with chimeric hPXRs. It might be because the space conformation of activation function helix [20] of hPXR had been changed by appending p53-AD to N- or C- termini of the hPXR, which resulted in its decreased sensitivity to RIF induction.

In C3A and HEK293T cells transiently transfected by chimeric hPXR constructs (**Fig. 2**), the response of CYP3A4.XREM.luc reporter gene activated by p53-hPXR expression vector was higher than that activated by hPXR-p53 expression vector. However, in C3A cells transiently transfected by hPXR-p53 construct, the expression level of CYP3A4 mRNA was higher than that by p53-hPXR construct (**Fig. 3**). A possible reason for this phenomenon may be because the space conformation of activation function helix of hPXR have changed with p53-AD appended to its C- termini, resulting in it being more conducive to transcription of CYP3A4 gene; on the other hand, other endogenous transcription factors involved in transcription of CYP3A4 gene may also be taken into account. The relevant underlying mechanism needs to be investigated further.

It needs to note that, compared to the increased level of CYP3A4 mRNA expression by chimeric hPXR transfection, the increase of induction expression of CYP3A4 mRNA seem not as much as expected, such as what can happen to primary hepatocytes. As we found such a trend of induction expression in both WT C3A and transfected C3A in the present work, we speculated that there might be mainly two reasons. One might be due to the cell itself – although the regulation activity of hPXR was enhanced by appending AD from p53 to it, C3A might still lack the other relevant component molecules which co-activated the induction reaction, thus the inducibility of transfected C3A was not improved effectively. Another possible reason could be that the concentration of RIF used in the induction experiment was not the most effective one for induction of CYP3A4 mRNA expression, as there was inducer-concentration dependency in such experiments using hepatic cells [33].

An earlier similar study performed by Küblbeck et al. [22] selected out two cell lines stably expressing chimeric hPXRs. Compared with WT C3A cells, the expression level of CYP3A4 mRNA increased 4.6 folds in the cell line with a higher expression of CYP3A4 and its metabolic activity increased 6.9 folds. By contrast, the CYP3A4 mRNA expression level in our A5 increased only 2.4 folds compared to WT C3A cells, but the protein level and metabolic activity of CYP3A4 in it appeared to be much better. This seemly inconsistent expression levels between mRNA and its protein product might attribute to the common phenomenon in cell biology that phenotypic behavior of cells depended not only on the expression of regulatory genes but also the relevant environmental factors. Culture conditions also have a clear effect on the enzyme activity in hepatoma cells [34]. For instance, certain liver-specific functions, including the metabolism by at least CYP1A2 and CYP3A4, are enhanced when cultivating hepatoma cells within three-dimensional alginate scaffolds, different co-culture systems or on PHBV microspheres to stimulate liver microarchitecture [23], [35], [36]. The CYP-mediated metabolism in hepatoma cells and primary human hepatocytes can be increased when adding chemicals, such as vitamin D, DMSO and dexamethasone to the culture media [8], [37], [38]. In addition, the confluency and culture time also affect the phenotype of cells, such as HepaRG, BC2 and B16A2 cells [5], [39], [40]. Thus, it is possible that modulation of the above processes could further improve the metabolic properties of the A5 cell line.

In conclusion, our results show that in hepatic cell line, the expression levels of CYP3A4 mRNA and protein and CYP3A4-mediated drug metabolism can be effectively enhanced by chimeric hPXR engineered with p53-AD. Taking this strategy, further studies will need to be carried out to improve the expression levels and activity of other enzymes or transporters under that same regulatory network.

## Supporting Information

Figure S1 **Agarose gel electrophoresis of the chimeric hPXR constructs digested by restriction endonucleases.** (A) 8 plasmids extracted from escherichia coli (E. coli) transformed by the hPXR-p53 construct were digested by *Nhe I* and *BamH 1*, then subjected to agarose gel electrophoresis. The Lane 2 and 6 were in accordance with our design, and then checked by DNA sequencing. (B) 4 plasmids extracted from E. coli transformed by the p53-hPXR construct were digested by *Nhe I* and *EcoR I*, then subjected to agarose gel electrophoresis. The Lane 2 and 3 were in accordance with our design, and then checked by DNA sequencing.
(TIF)

Figure S2 **The sequencing result of the chimeric fragment hPXR-p53.**
(TIF)

Figure S3 **The sequencing result of the chimeric fragment p53-hPXR.**
(TIF)

Figure S4 **PXR mRNA expression in WT C3A and modified C3A cells stably transfected with pCI-hPXR-p53 construct (A1–A7) or pCI-p53-hPXR construct (B1–B6).** The relative mRNA levels compared with mRNA levels in the WT C3A without the addition of RIF control cells (relative expression value set to 1) were defined by the 2^−ΔΔCT^ method. Results are expressed as the mean±S.D. of normalized expression, n = 3.
(TIF)

Figure S5 **GADD45A and BAX mRNAs expression in WT C3A and modified C3A cells stably transfected with pCI-hPXR-p53 construct (A1–A7) or pCI-p53-hPXR construct (B1–B6).** The relative mRNA levels compared with mRNA levels in the WT C3A without the addition of RIF control cells (relative expression value set to 1) were defined by the 2^−ΔΔCT^ method. Results are expressed as the mean±S.D. of normalized expression, n = 3.
(TIF)

Figure S6 **CYP2B6 mRNA expression in WT C3A and C3A cells stably transfected with chimeric hPXRs.** The relative mRNA levels compared with mRNA levels in the WT C3A without the addition of RIF control cells (relative expression value set to 1) were defined by the 2^−ΔΔCT^ method. Results are expressed as the mean±S.D. of normalized expression, n = 3. Statistical significance p<0.05: *, RIF-treated vs. respective DMSO control; ***, C3A cells stably transfected with chimeric hPXRs vs. WT C3A.
(TIF)
