# Supplementary figures and images for: Up-Regulating CYP3A4 Expression in C3A Cells by Transfection with a Novel Chimeric Regulator of hPXR-p53-AD
Source: PLoS One. 2014 May 1;9(5):e95752. doi: 10.1371/journal.pone.0095752 (PMC4006776; doi:10.1371/journal.pone.0095752)

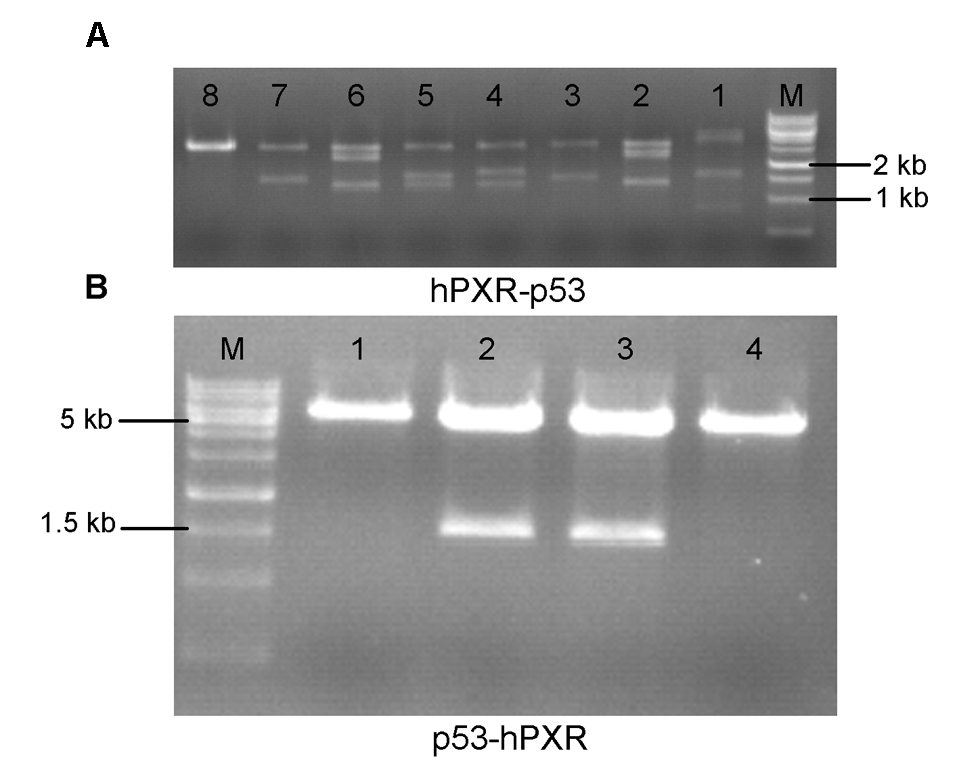

Supplement: Figure S1 — Agarose gel electrophoresis of the chimeric hPXR constructs digested by restriction endonucleases. (A) 8 plasmids extracted from escherichia coli (E. coli) transformed by the hPXR-p53 construct were digested by Nhe I and BamH 1, then subjected to agarose gel electrophoresis. The Lane 2 and 6 were in accordance with our design, and then checked by DNA sequencing. (B) 4 plasmids extracted from E. coli transformed by the p53-hPXR construct were digested by Nhe I and EcoR I, then subjected to agarose gel electrophoresis. The Lane 2 and 3 were in accordance with our design, and then checked by DNA sequencing. (TIF) [file pone.0095752.s001.tif]

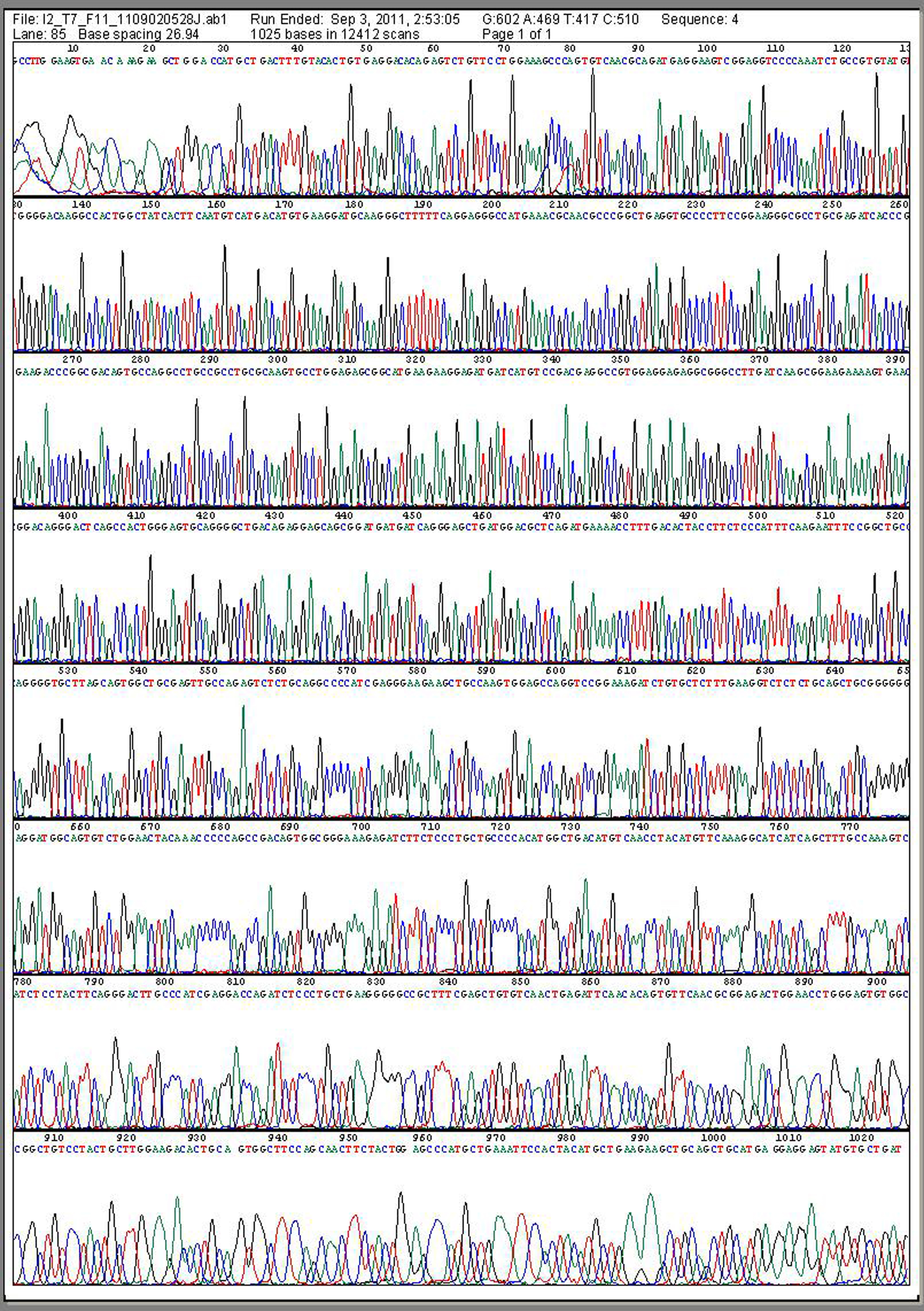

Supplement: Figure S2 — The sequencing result of the chimeric fragment hPXR-p53. (TIF) [file pone.0095752.s002.tif]

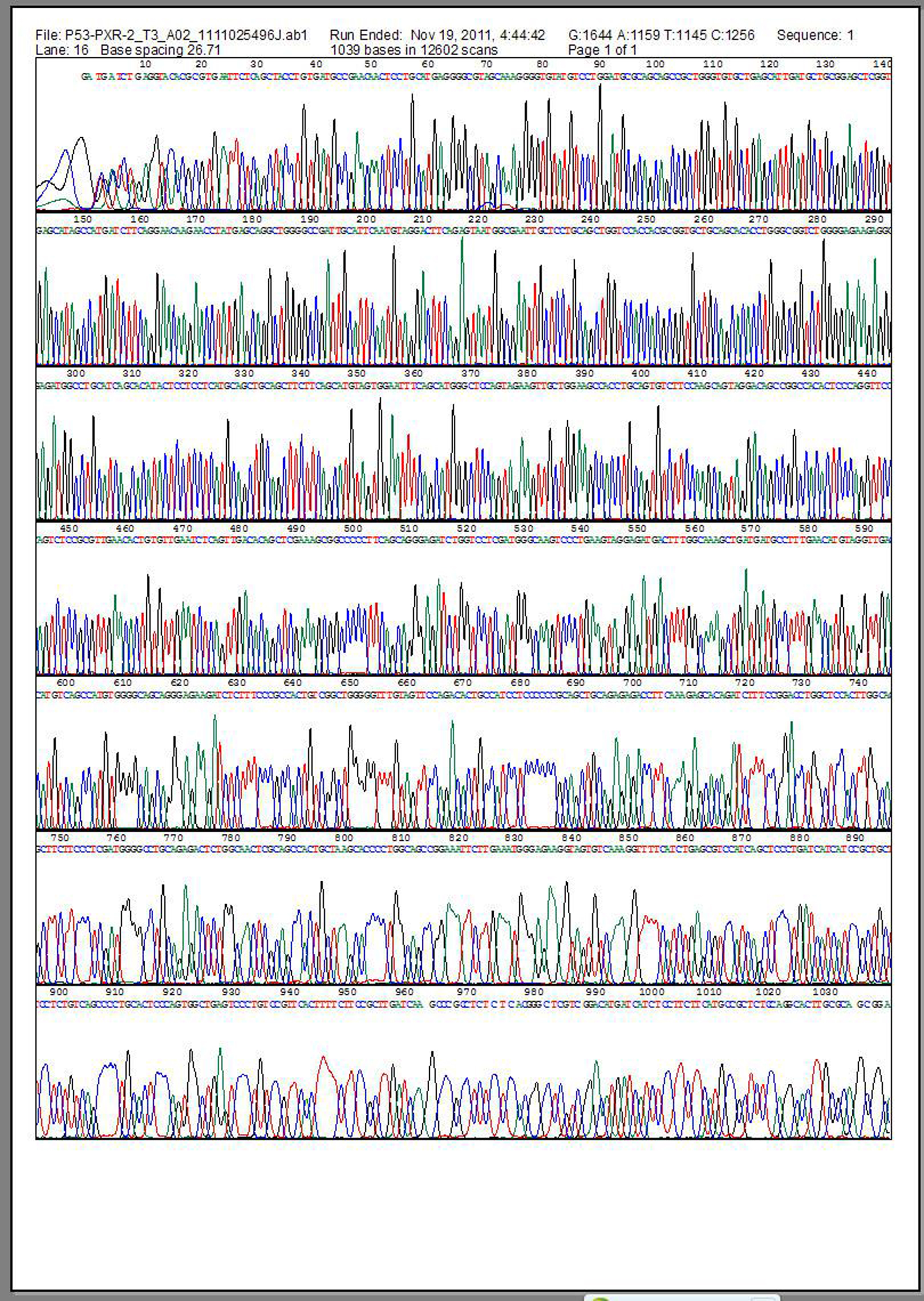

Supplement: Figure S3 — The sequencing result of the chimeric fragment p53-hPXR. (TIF) [file pone.0095752.s003.tif]

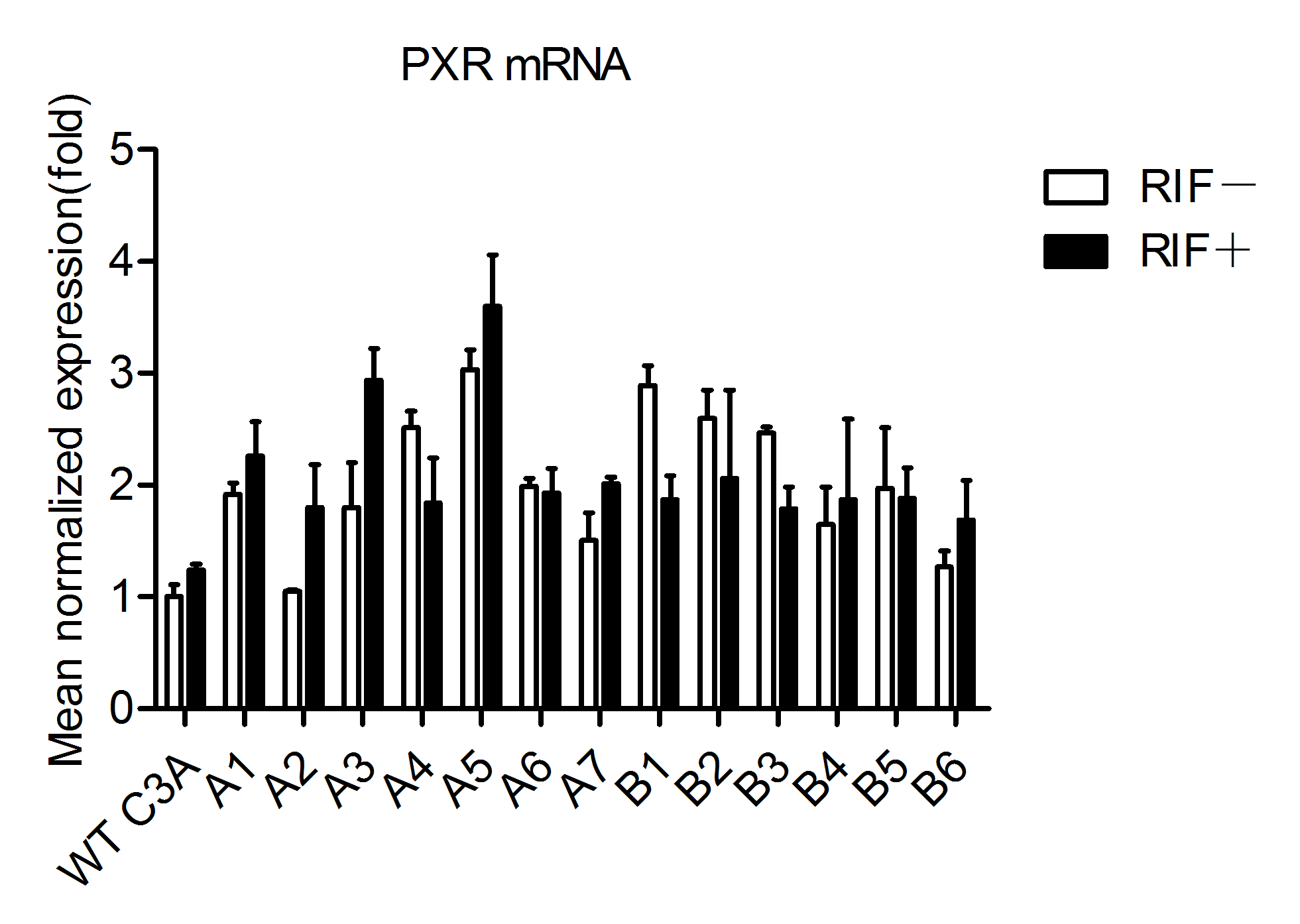

Supplement: Figure S4 — PXR mRNA expression in WT C3A and modified C3A cells stably transfected with pCI-hPXR-p53 construct (A1–A7) or pCI-p53-hPXR construct (B1–B6). The relative mRNA levels compared with mRNA levels in the WT C3A without the addition of RIF control cells (relative expression value set to 1) were defined by the 2−ΔΔCT method. Results are expressed as the mean±S.D. of normalized expression, n = 3. (TIF) [file pone.0095752.s004.tif]

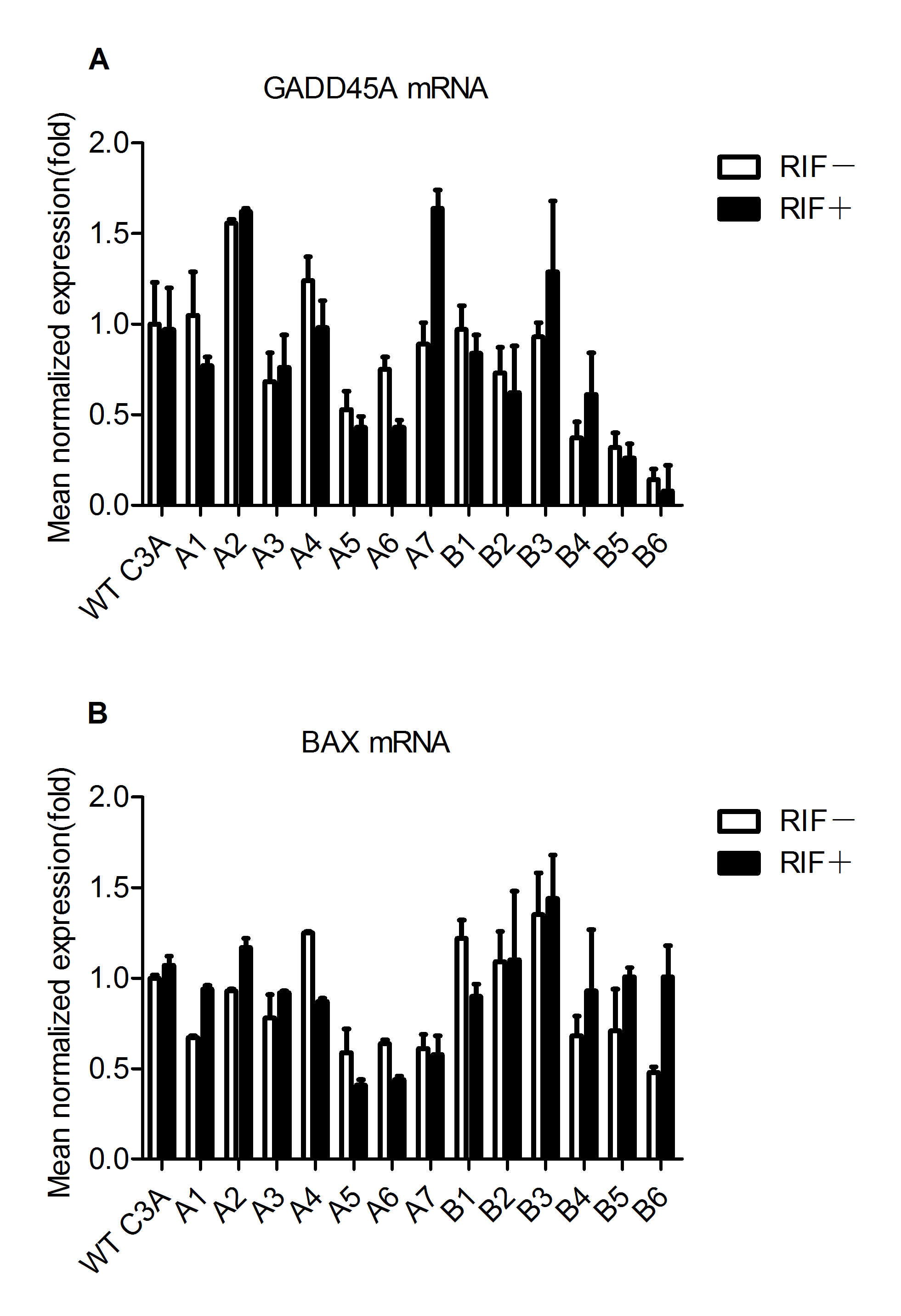

Supplement: Figure S5 — GADD45A and BAX mRNAs expression in WT C3A and modified C3A cells stably transfected with pCI-hPXR-p53 construct (A1–A7) or pCI-p53-hPXR construct (B1–B6). The relative mRNA levels compared with mRNA levels in the WT C3A without the addition of RIF control cells (relative expression value set to 1) were defined by the 2−ΔΔCT method. Results are expressed as the mean±S.D. of normalized expression, n = 3. (TIF) [file pone.0095752.s005.tif]

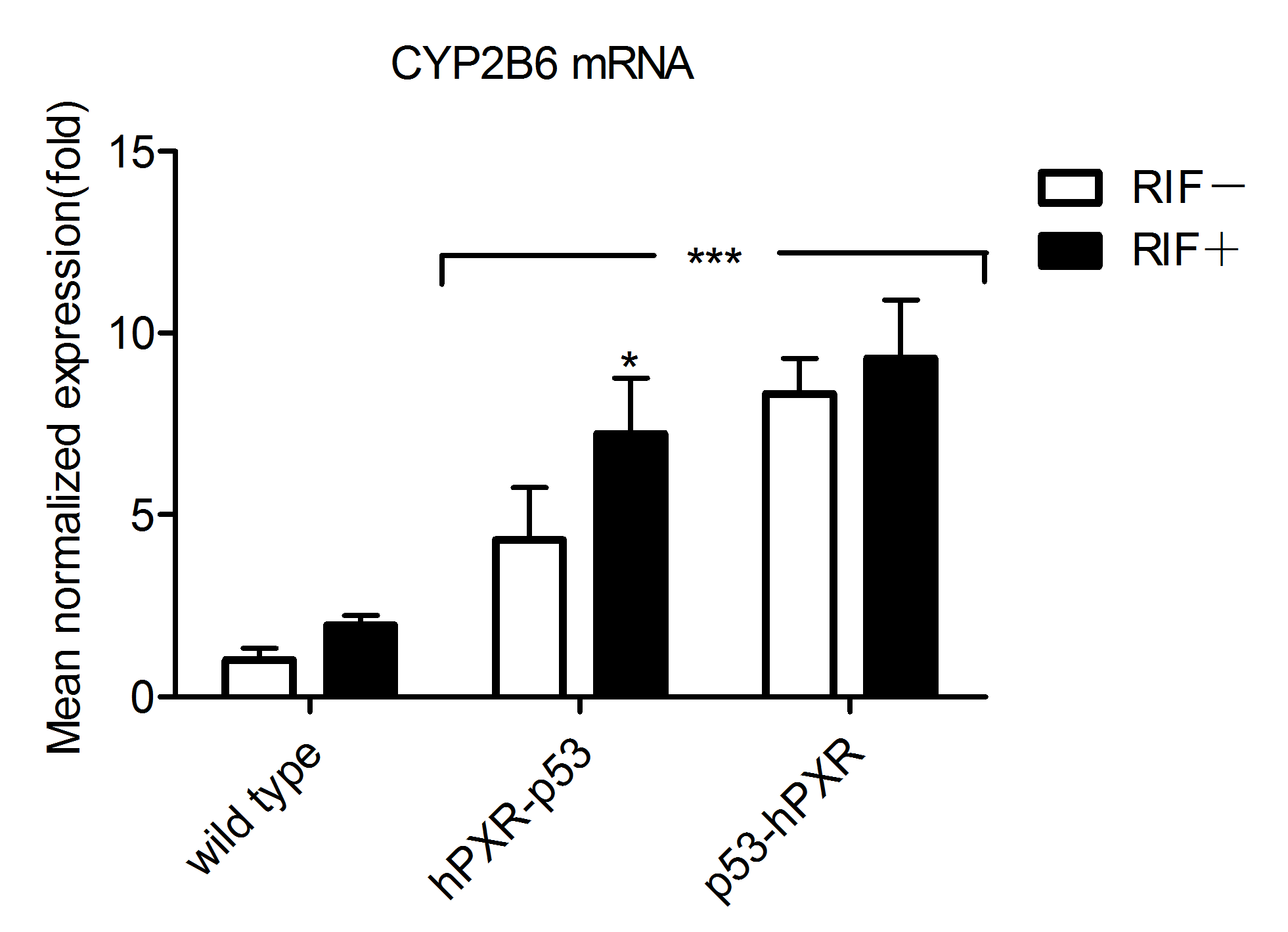

Supplement: Figure S6 — CYP2B6 mRNA expression in WT C3A and C3A cells stably transfected with chimeric hPXRs. The relative mRNA levels compared with mRNA levels in the WT C3A without the addition of RIF control cells (relative expression value set to 1) were defined by the 2−ΔΔCT method. Results are expressed as the mean±S.D. of normalized expression, n = 3. Statistical significance p<0.05: *, RIF-treated vs. respective DMSO control; ***, C3A cells stably transfected with chimeric hPXRs vs. WT C3A. (TIF) [file pone.0095752.s006.tif]
